# Femtosecond Laser-Written Nanoablations Containing Bright Antibunched Emitters on Gallium Nitride

**DOI:** 10.1021/acsphotonics.5c01506

**Published:** 2025-10-02

**Authors:** Yanzhao Guo, Giulio Coccia, Vibhav Bharadwaj, Reina Yoshizaki, Katie M. Eggleton, John P. Hadden, Shane M. Eaton, Anthony J. Bennett

**Affiliations:** † School of Engineering, 2112 Cardiff University, Queen’s Buildings, The Parade, Cardiff CF24 3AA, United Kingdom; ‡ Department of Physics, 274268 Politecnico di Milano, Piazza Leonardo da Vinci, 32, 20133 Milano, Italy; § Department of Physics, Indian Institute of Technology Guwahati, 781039 Guwahati, Assam, India; ∥ Department of Mechanical Engineering, School of Engineering, 13143 The University of Tokyo, Hongo, Bunkyo-ku, Tokyo 113-8656, Japan; ⊥ Translational Research Hub, Cardiff University, Maindy Road, Cardiff CF24 4HQ, United Kingdom; # Institute for Photonics and Nanotechnologies (CNR-IFN), Piazza Leonardo da Vinci, 32, 20133 Milano, Italy

**Keywords:** semiconductor, quantum light, single photon, color center, defects

## Abstract

Femtosecond laser-writing offers distinct capabilities for fabrication, including three-dimensional, multimaterial, and sub-diffraction-limited patterning. In particular, demonstrations of laser-written quantum emitters and photonic devices with superior optical properties have attracted attention. Recently, gallium nitride (GaN) has been reported to host quantum emitters with narrow and bright zero-phonon photoluminescence from ultraviolet to telecom ranges. However, emitters formed during epitaxy are randomly positioned, and until now, it has not been possible to fabricate quantum emitters in ordered arrays. In this paper, we employ femtosecond laser writing to create nanoablations with sub-diffraction-limited diameter and use rapid thermal annealing to activate co-located stable emitters. The emitters show a MHz antibunched emission with a sharp spectral peak at room temperature. Our study not only presents an efficient approach to laser-written nanofabrication on GaN but also offers a promising pathway for the deterministic creation of quantum emitters in GaN, shedding light on the underlying mechanisms involved.

## Graphical abstract

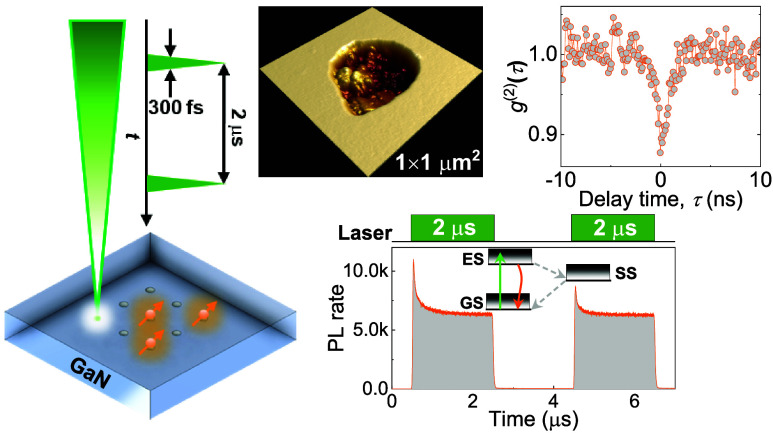

## Introduction

In the past decade, quantum emitters (QEs) in gallium nitride (GaN) have been investigated as a practical source of quantum light at room temperature.^1^ These QEs display low multiphoton emission probability,^2^ comparatively high Debye–Waller factor,^3^ high continuous-wave (CW) photon detection rate,^4^ and telecom range emission.^5^ Recently, resonant optical excitation^6^ and optically detected magnetic resonance have been observed in GaN QEs,^3,7^ pointing to potential applications in quantum sensing and quantum networks. However, the reliable fabrication of QEs in GaN remains a challenge. This greatly hinders high-yield coupling to photonic structures that manipulate the local density of optical states and enhance photon collection,^8^ which is crucial for the realization of scalable integrated quantum photonics.

Previous approaches to creating these QEs, such as ion implantation and doping during growth,^2,3,9–11^ create QEs with random positions and residual lattice damage^12,13^ which one may expect to degrade the spin and optical coherence properties. Recently, femtosecond laser-writing fabrication has attracted intense attention for its precise and highly controlled creation of QEs in semiconductors, such as diamond,^14–16^ silicon carbide,^17^ hexagonal boron nitride,^18,19^ and aluminum nitride.^20^ Moreover, femtosecond laser-writing is also used as a promising method to fabricate three-dimensional (3D) photonic circuits from nanoscale to microscale.^12,13,21^ Direct laser-writing offers a maskless fabrication process below the optical diffraction limit. Under optimal fabrication conditions, laser-written waveguide-integrated QEs in diamond feature spin coherence properties comparable to their native counterparts in the host material.^22,23^

In this paper, we report femtosecond laser-writing and subsequent annealing to fabricate an array of emitters in GaN. Because the bright spots observed in the photoluminescence (PL) scan are in a regular square array, we can be certain that the laser writing induces them and is not intrinsic to the semiconductor. As we will show, bright emission is correlated in position with the nanoablated holes on the sample surface. Laser-written nanoablations in GaN are mapped by atomic force microscopy (AFM) and confocal photoluminescence (PL) mapping. Compared to previously studies which reported broadband PL emission,^24,25^ the laser-written structures in this paper exhibit a sharp emission peak in their spectrum at room temperature, antibunching in their photon emission correlation spectra (PECS), shelving behavior in time-resolved PL (TRPL) measurements, and few MHz saturation PL rates under CW excitation. These features are consistent with the creation of a small number of QEs in a sub-diffraction-limited spot. Our study provides a promising route to scalable, integrated, on-chip quantum technologies based on GaN via femtosecond laser writing.

## Results and Discussion

### Femtosecond Laser-Writing Fabrication on GaN

The sample used in this experiment is a free-standing single-crystal nominally undoped GaN substrate with a (0001) C-plane orientation. As shown in Figure 1(a), a commercial femtosecond laser (Menlo Systems BlueCut) that produces linearly polarized pulses with a wavelength of 515 nm, a repetition rate of 500 kHz, and a duration of 300 fs was used for femtosecond laser-writing. A 100× oil immersion objective with a numerical aperture (NA) of 0.9 is used to focus the laser beam in GaN. The pulse energy was controlled using a combination of a motorized half-wave plate and a fixed linear polarizer. There are four different laser fabrication sets with laser exposure pulse numbers of *N* = 1, 2, 5, and 10, respectively. As shown in Figure 1(b), in each set, the laser energies are spaced with 20 μm from *E* = 5 to 510 nJ, where there are 5 trials for each laser-writing parameter.

**Figure 1:**
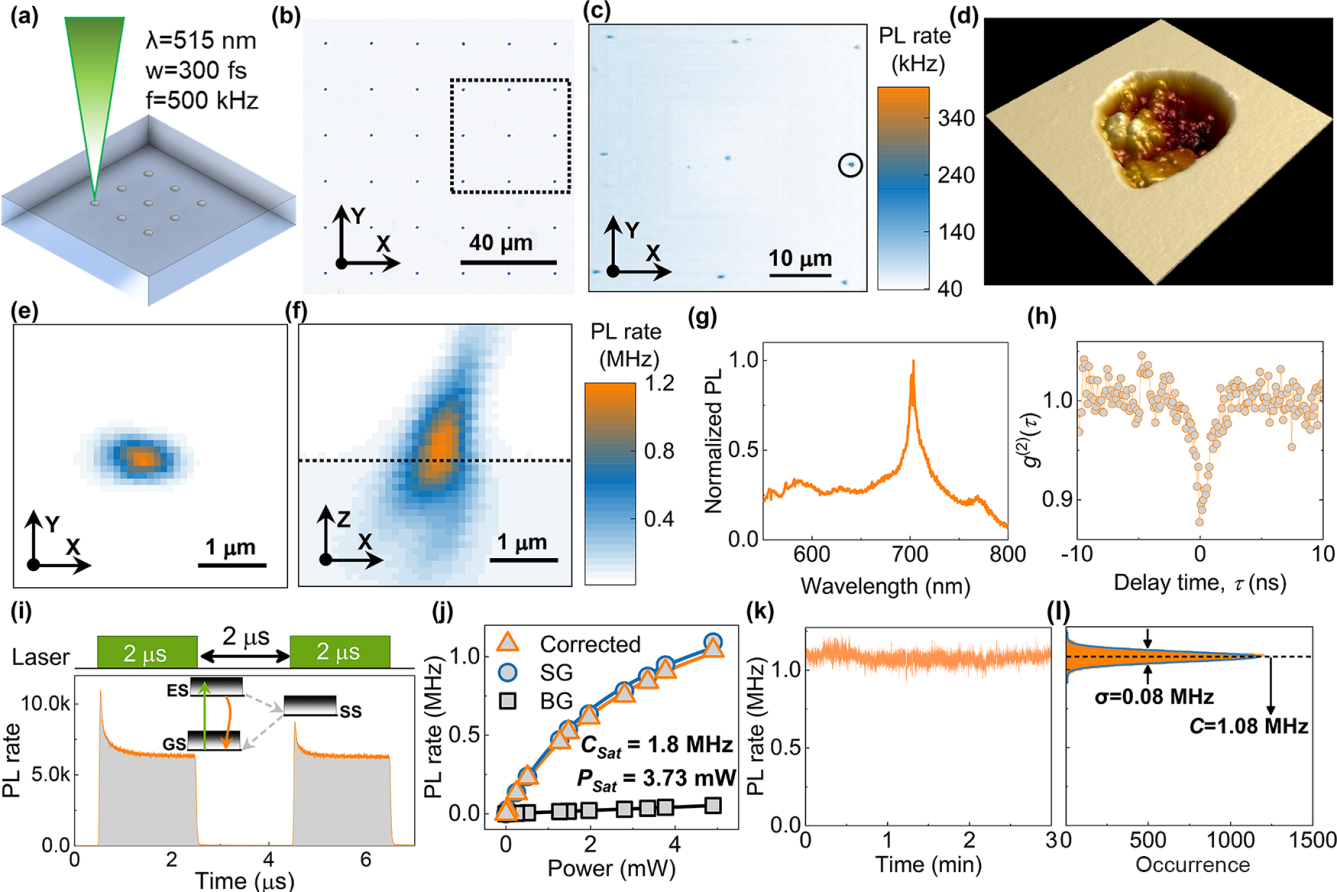
Laser-written antibunched emitters in GaN. (a) Schematic diagram of laser-writing fabrication on GaN. (b) An optical microscope image for laser-written GaN, where the area marked by a rectangular dashed box is inspected by a PL scanning of (c, d) is the 1 μm × 1 μm AFM image for the laser-written nanoablation marked in (c, e) and (f) are the *xy* and *xz* PL maps for the laser-written emitter marked in (c), where the horizontal black dashed line in (f) represents the GaN surface. (g, h) Normalized PL emission spectrum and photon emission correlation spectrum for this laser-written emitter. (i) Time-resolved PL spectrum under double-pulse laser excitation, with the inset showing a three-energy-level system. (j) Power-dependent PL saturation measurement. (k) PL time trace of the emitter sampled into 10 ms bins under CW laser excitation. (l) Corresponding histogram of the photon distribution.

### Laser-Written Bright Antibunched Emitters

The AFM scanning image in Figure 1(d) shows that the femtosecond laser produces a nanoablation with a rough interior. The AFM scanning measurement details are in the Experimental Methods section. Our hypothesis is that high-energy femtosecond laser results in strong nonlinear absorption in GaN,^12^ which breaks the GaN lattice bond and leaves a nanoablation.^21^ After annealing, there is no emission in the laser-written areas, where the fabrication laser energy is below the ablation threshold. In contrast, an array of emitters is observed around the laser-written region where the laser energy is over the threshold, as shown in Figure 1(c). The *xy* PL map of Figure 1(e) and the *xz* PL map of Figure 1(f) reveal a MHz-rate point-like emission at this location. The PL emission spectrum in Figure 1(g) shows a sharp peak at around 702 nm. In general, the emitters created in this study have spectral peaks in the range 550–800 nm with room-temperature line widths over 10 nm, which is similar to previously reported emitters formed during epitaxy on sapphire.^3,4,10^ We take these peaks to correspond to the zero-phonon line of each emitter. We note that the majority of nanoablations contain many emission peaks, suggesting the presence of more than one emitter. However, previous studies of laser-written defects buried in GaN have only shown broadband emission, consistent with the presence of a large number of emitters.^24,25^ The transitions are substantially broader than the lifetime-limited line widths, which is common for room-temperature quantum emitters as a result of phonon- and charge-induced broadening, and may be further broadened by the presence of more than one emitter per site. PECS data, recorded by two detectors in a Hanbury-Brown and Twiss interferometer, in Figure 1(h) shows antibunching behavior with *g* ^(2)^(0) < 0.9, consistent with the creation of multiple quantum emitters within the nanoablation.

To understand these emitters’ bunching behavior and energy structure,^26^ a TRPL spectrum is recorded under two 2 μs duration laser pulse excitation with a 2 μs space, as shown in Figure 1(i). The spacing between each double-pulse train is 50 μs to allow the ground state population to reset. From the shelving behavior during the laser pulse excitation, we deduce that at least three energy levels are present in these emitters, which includes the ground state (GS), excited state (ES), and shelving state (SS), consistent with the previous study.^27^ In Figure 1(j), the excited power-dependent PL intensity is fitted by

1 $$C\left(P\right)=\frac{C_{\mathrm{sat}}P}{P+P_{\mathrm{sat}}}$$

where *C*(*P*) is the steady-state PL rate as a function of power *P*, *C*_sat_ is the saturation PL rate, and *P*_sat_ is the corresponding saturation power. A saturation PL rate of 1.8 MHz is achieved with 3.73 mW of saturation power. We also recorded the time trace of this emitter’s PL rate for 3 min, where the emission shows a mean count rate of 1.08 MHz and displays a variance of 0.08 MHz.

### Laser-Writing Parameters for Nanoablations with Quantum Emitters

A statistical study of these laser-written nanoablations is performed by comparing their AFM images and PL emission. First of all, from their AFM images in Figure 2(a) and cross section in Figure 2(b), both increased laser energies and pulse numbers result in increased depth and diameter of the laser-written nanoablations. Second, compared to laser energies, laser pulse numbers have a strong impact on the roughness of these nanoablations. Specifically, the nanoablations written by laser pulse number *N* = 1, 2 exhibit smooth interface, while the nanoablations fabricated by laser pulse number *N* = 5, 10 feature relatively rough morphology, as shown in the cross sections displayed in Figure 2(b). Third, Figure 2(c) shows the yield of emitters in the nanoablations increased as laser pulse numbers increased. Less than 4% of the *N* = 1 laser-written nanoablations show resolved PL emission. For *N* = 2, we find 33% of nanoablations display emission, and for *N* = 5 and *N* = 10, over 97%. Finally, we note that laser energies show a weak relation to the PL rate of laser-written emitters. In addition, emitters written with *N* = 1,2 pulses exhibit broadband emission over 550–800 nm without a resolvable ZPL at room temperature, whereas *N* = 5,10 pulses yield a similar broadband background with narrow peaks in this range, and no systematic dependence on laser-writing energy was observed. These observations lead us to conclude that the increased laser pulse number results in complicated surface structures of these nanoablations, which provide the emissive sources and/or the structural defect that leads to the emission. Additionally, a single shot of femtosecond laser creates a laser-written nanoablation with a smoother surface. At single pulse laser energies less than 260 nJ, the nanoablation’s diameter and depth will be reduced to ∼100 and ∼45 nm, which is less than half of the optical diffraction limit. This precise positioning ability potentially paves the way for efficient direct laser-written nanoholes in GaN coupled to photonic structures.^28^

**Figure 2:**
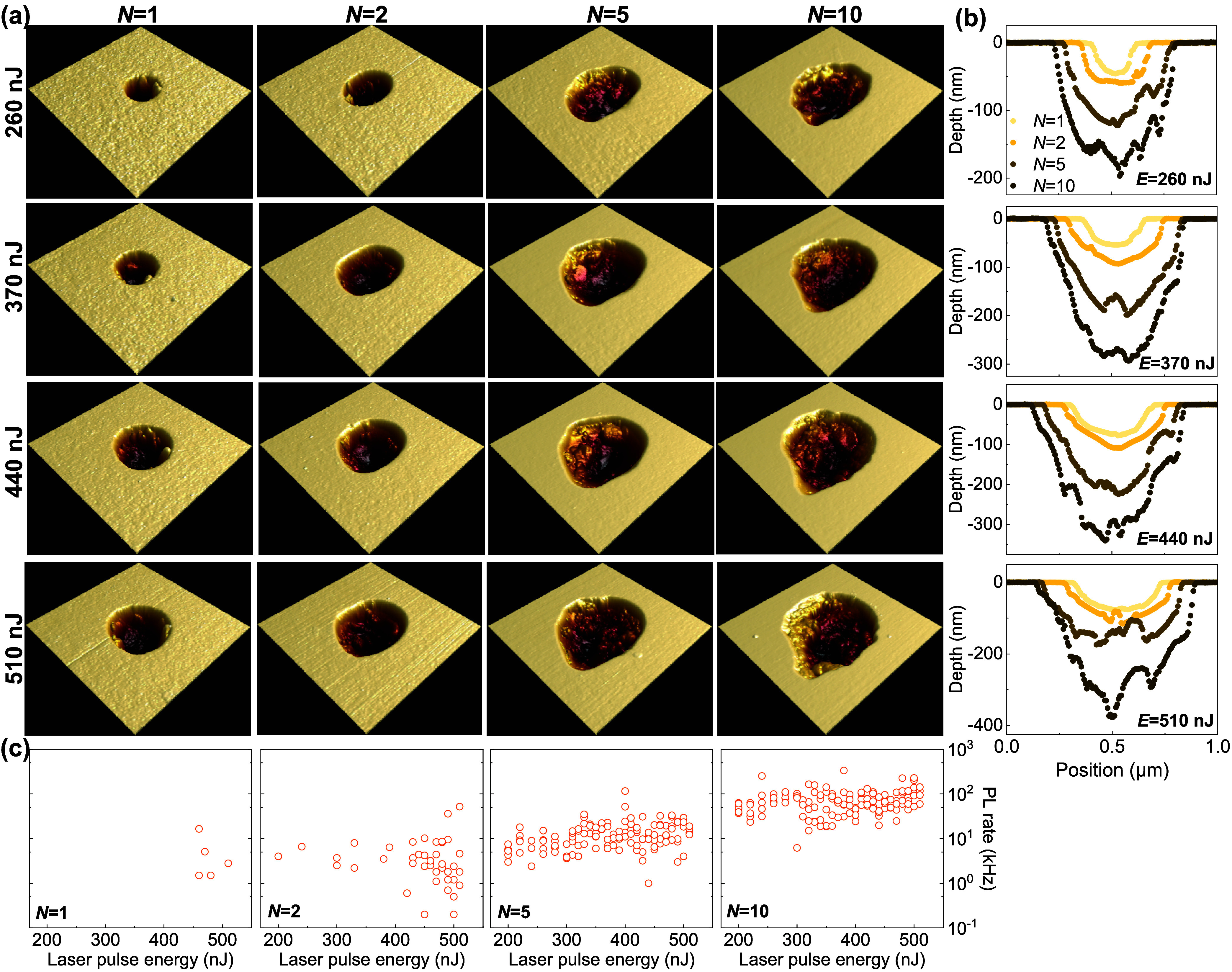
AFM images and yield analysis for the emitters in laser-written nanoablations in GaN. (a) 1 mm × 1 mm AFM images of the laser-written nanoablations with different energies (rows) and pulse numbers (columns). (b) Cross sections from AFM images for different laser pulse numbers and different laser energies (columns). (c) PL emission of laser-written emitters in different pulse numbers as a function of laser-writing energies.

### Photodynamics Study on Laser-Written Emitters in GaN

We investigate the photodynamics of these laser-written emitters by TRPL and PECS. Based on the TRPL in Figure 3(a). All emitters feature a PL peak at the start of the first laser pulse, as shown in Figure 3(a). In contrast, the background signal intensity linearly scales with the laser power and shows the same temporal profile as the excitation laser, corresponding to the rise of the signal from the AOM (Figure 3(a), top panel). Emitter E1 has the weakest PL emission and does not display strong shelving decay in its TRPL. This type of behavior is mostly observed in *N* = 1 and 2 laser pulse-written areas. Emitter E2 emits a stronger PL signal, features an additional shelving decay within hundreds of nanoseconds in its TRPL, and is found in laser-written regions with *N* = 5, 10. Emitter E3 features MHz PL rate and exhibits tens of ns shelving process. Approximately 5% of the nanoablations with laser pulse number *N* = 10 are of this type. In the time between laser pulses, the system does not fully relax to the ground state in E2 or E3, resulting in a lower amplitude intensity at the start of the second pulse. This is indicative of a several μs shelving state lifetime.^26^

**Figure 3:**
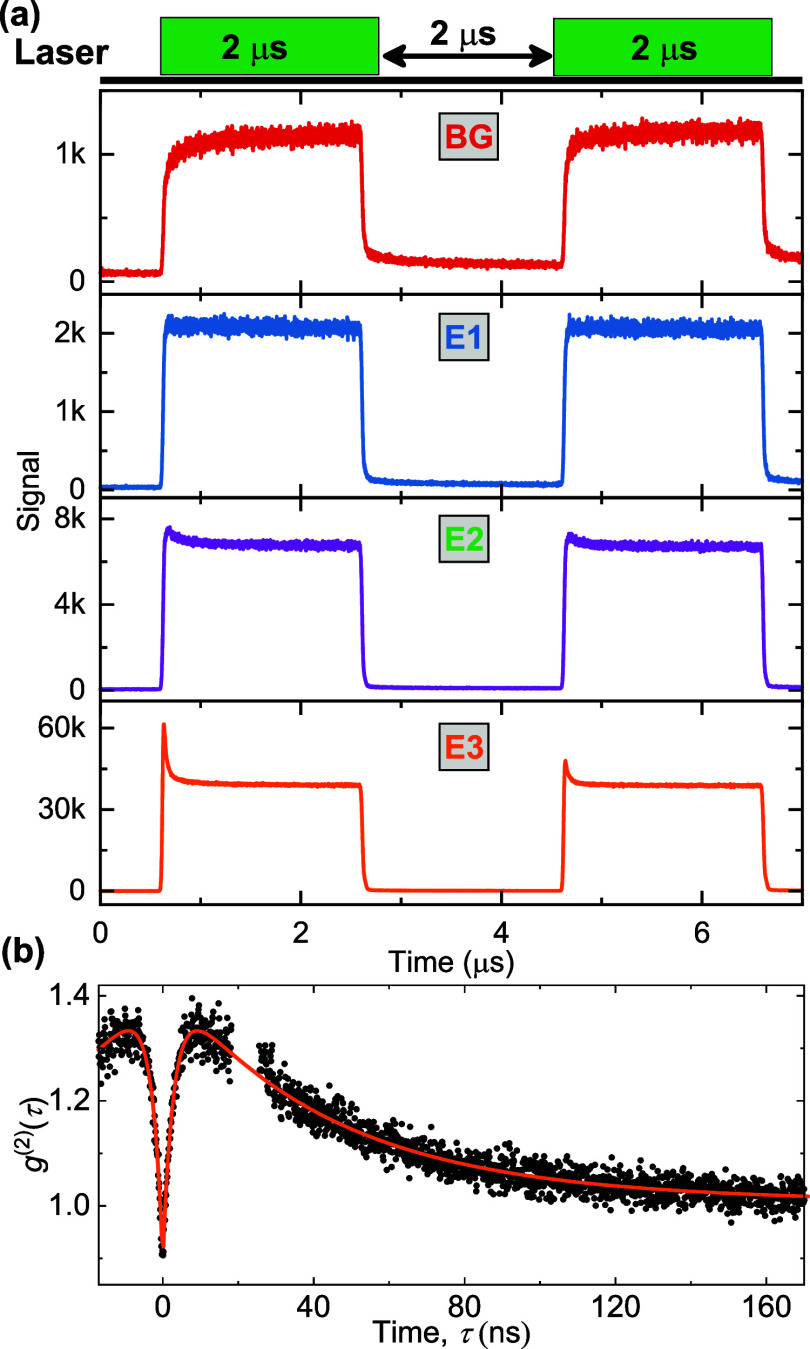
Photodynamics study of laser-written emitters in GaN. (a) TRPL study for different emitters and background under double-pulse excitation. (b) PECS for E3.

Less intense emitters like E1 and E2 did not exhibit antibunching or bunching in their PECS. In contrast, E3 shows an antibunching signal in its PECS in Figure 3(b). The *g* ^(2)^(τ) data is fitted using the empirical equation

2 $$g^{\left(2\right)}\left(\tau \right)=1-C_{1}e^{-\left|\tau \right|/\tau_{1}}+C_{2}e^{-\left|\tau \right|/\tau_{2}}+C_{3}e^{-\left|\tau \right|/\tau_{3}}$$

Here, τ_1_ is the antibunching time, *C*_1_ is the antibunching amplitude, τ_*i*_ for *i* ≥ 2 are bunching times, and *C*_*i*_ for *i* ≥ 2 are the corresponding bunching amplitudes. The fitting parameters are listed in Table 1.

**Table 1:** Fitting Parameters of *g* ^(2)^(τ)

parameters	value	standard error
*C*_1_	0.54	0.005
τ_1_ (ns)	2.90	0.06
*C*_2_	0.42	0.003
τ_2_ (ns)	42.93	0.59
*C*_3_	0.019	0.002
τ_3_ (ns)	290.84	56.46

Overall, the PL emission of QEs is defined by its excited state lifetime and shelving process.^26,29^ For the TRPL measurement, the time trace is partly determined by the population in the ground state at the beginning of the laser pulse. Therefore, E3 exhibits a sharp rise at the beginning of laser pulses due to the laser-pumped occupation of its excited state and subsequently follows an exponential decay until a steady state is reached due to the shelving process. This also results in the bunching behavior of E3 in Figure 3(b). In conclusion, our photodynamic study results indicate that the shelving process prevails in laser-written emitters, confirming the dynamics of emission from their atomic-like energy levels.

### Annealing Study

Before annealing, the laser-written ablations are difficult to resolve from the PL scanning, as shown in Figure S1­(a). Only a few laser-written ablations emit weak PL emission above the background level. These all show dipole-like excitation polarization dependence and a broadband PL spectrum from 550 to 800 nm without the antibunching (Figure S1). Thus, these laser-written regions might be some optically active structural dislocation that cannot form a stable QE without further annealing or there may be many emitters within the confocal laser spot. Subsequently, a series of rapid thermal annealing (RTA) processes are used to activate the laser-written emitters in a nitrogen gas ambiance. After the first 0.5 h of 400 °C annealing, the laser-written areas become brighter and show weak dependence on laser-written energies, with an improved PL contrast compared to the background, as shown in Figure S2. Most of the laser-written emitters show properties similar to those of the emitters before annealing, exhibiting dipolar-like excitation polarization, a broadband spectrum from 550 to 800 nm. Some blinking emitters are also found on the edge of laser-written nanoablation, as circled in Figure S2­(a), and close to the surface of the substrate, as shown in Figure S2­(b). More importantly, they show antibunching in their PECS. However, these QEs are highly unstable and suffer from photobleaching, which makes them unable to collect their spectra.

After the second 0.5 h of 400 °C annealing, an increasing number of blinking QEs are observed in the laser-written region, some with weak antibunching (*g* ^(2)^(0) ≈ 0.9). Their emission is broad between 550 and 800 nm but with a few sharp peaks. Their power-dependent PL shows saturation behavior. After the third 0.5 h of 400 °C annealing, we found some stable and bright emitters with MHz of PL rate around the laser-written region, such as E3 in Figure 3.

We monitor E3 over several further cycles of annealing, showing substantial changes to the saturation rate and spectrum in Figure 4. There are two peaks around 600 and 700 nm, whose intensities are affected via annealing. This highlights the dynamic processes of creation and annihilation for different species of QEs in laser-written spots. After three anneals of 0.5 h at 400 °C, two anneals of 0.5 h at 500 °C, and one anneal of 0.5 h at 600 °C, a sharper peak is resolved at a wavelength of 600 nm. However, after the second 0.5 h at 600 °C, these sharp peaks are suppressed by the strong phonon sideband, exhibiting the unstable PL emission with photobleaching.

**Figure 4:**
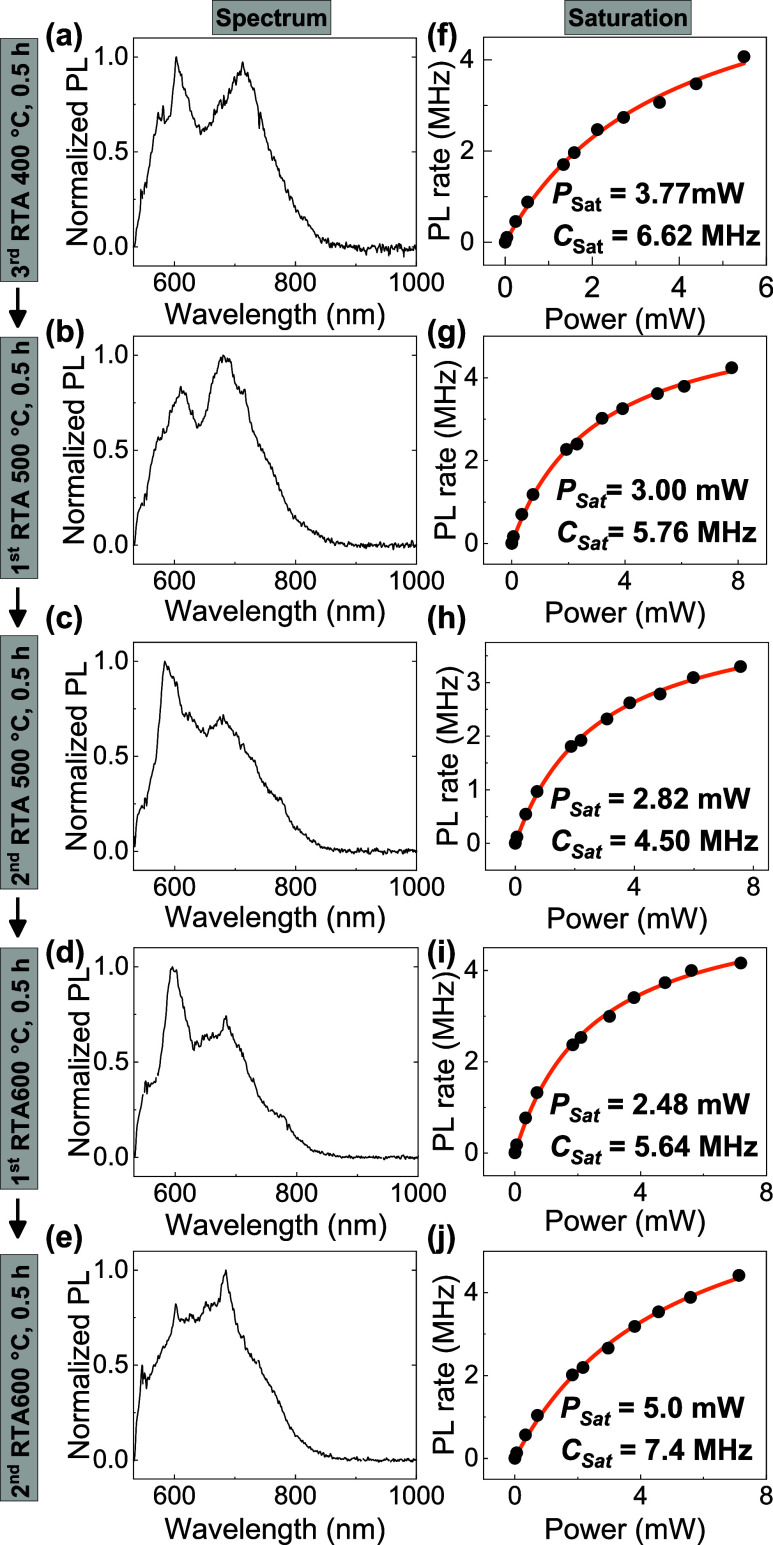
Normalized spectra (a–e) and power-dependent PL saturation behaviors (f–j) for E3 during the RTA annealing.

Another signature of a quantized emitter is its photoluminescence (PL) intensity saturation with excitation. From Figure 4(f–j), the *P*_sat_ decreases with the annealing process until the second time 0.5 h of 600 °C annealing, and then increases again as new features appear in the spectrum for subsequent anneals. These results suggest that 0.5 h of 600 °C RTA annealing is a threshold for the creation of stable quantum emitters. This may be related to a previous annealing study,^30^ which predicted that diffusion of nitrogen vacancy centers occurs at above 500 °C.

## Conclusions and Outlook

We report the engineering of a regularly spaced array of QEs in GaN via femtosecond laser-writing and subsequent annealing. Bright, antibunched emission with MHz PL rates and sharp spectral peaks are deterministically created in the laser-written nanoablations. The laser-writing effect is also investigated as a function of laser energy, pulse number, and annealing using AFM and PL studies. We find that laser-written nanoablations with an increased laser pulse number (*N* = 5, 10) annealed at 500–600 °C are optimal to create emitters that display the signatures of quantized electronic states. We were not able to determine a trend in emission wavelength with pulse number or energy, but this could be investigated in future samples with a larger number of nanoablations.

Our study paves a promising way to scalable engineering of QEs inside photonic nanostructures and integrated quantum circuits. Future works should focus on increasing the yield of bright laser-written emitters and fabricating the emitters at a single level, possibly by using in situ laser annealing on individual sites combined with real-time feedback of emitter creation, as shown in negatively charged nitrogen vacancy centers formation in diamond by Chen et al.^15^ Alternatively, low-temperature spectroscopy of multiple emitters in a single site may allow spectral isolation of single transitions, which would also demonstrate a higher degree of antibunching than that reported here. Moreover, our study also demonstrates an efficient direct laser nanoablation process for GaN, providing important information for the fabrication of laser-written photonic circuits.

## Experimental Methods

### Confocal Setup

A home-built room-temperature confocal setup is used to study the PL emission for the laser-written GaN sample. A CW 532 nm crystal laser was modulated by an acoustic-optic modulator (ISOMET 553F-2) with <10 ns rise and fall time. Time-resolved PL spectrum was binned with 1 ns resolution. A 2-axis Galvo mirror (GVS002) and 100× Nikon objective with NA = 0.9 were integrated into a 4f imaging system for 2D *x*–*y* scanning. Depth scanning (*z*) was implemented by a closed-loop piezo sample stage. The PL was optically filtered by the dichroic mirror, 532 nm long-pass filter before detection on SPCM-AQRH silicon avalanche photodiodes (Excelitas) or a spectrometer with a silicon CCD. The optional ND filter is also used to keep the PL rate within the APD’s linear response range (2 MHz).

### AFM Measurement

The AFM measurement was conducted with commercial Bruker AFM microscopes. The scanning tip is the silicon tip on the nitride lever. The scan parameters are 1 Hz of scanning rate with 256 samples and 0° of scan angle. The AFM image was analyzed and plotted by the Bruker commercial software (Nanoscope Analysis 3.0).

## Supplementary Material



## Data Availability

Data supporting the findings of this study are available in the Cardiff University Research Portal at http://doi.org/10.17035/cardiff.28883237.

## Notes

Photoluminescence maps of laser-written nanoablation in gallium nitrides before and after the first 0.5 h of 400 °C (PDF)
