# Supplementary material for: Femtosecond Laser-Written Nanoablations Containing Bright Antibunched Emitters on Gallium Nitride
Source: ACS Photonics. 2025 Oct 2;12(10):5716–22. doi: 10.1021/acsphotonics.5c01506 (PMC12532371; doi:10.1021/acsphotonics.5c01506)
Supplement: Supplementary file 1 [file ph5c01506_si_001.pdf]

Supporting Information for Femtosecond laser-written nano-ablations containing bright antibunched emitters on gallium nitride

Yanzhao Guo,[*,†,||] Giulio Coccia,[‡,⊥] Vibhav Bharadwaj,[¶,⊥] Reina Yoshizaki ,[§] Katie M. Eggleton,[†,||] John P. Hadden,[†,||] Shane M. Eaton,[‡,⊥] and Anthony J. Bennett[*,†,||]

†*School of Engineering, Cardiff University, Queen's Buildings, The Parade, Cardiff, CF24 3AA, United Kingdom*

‡*Department of Physics, Politecnico di Milano, Piazza Leonardo da Vinci, 32, 20133 Milano, Italy*

¶*Department of Physics, Indian Institute of Technology Guwahati, 781039 Guwahati, Assam, India*

§*Department of Mechanical Engineering, School of Engineering, The University of Tokyo, Hongo, Bunkyo-ku, Tokyo 113-8656, Japan*

||*Translational Research Hub, Cardiff University, Maindy Road, Cardiff, CF24 4HQ, United Kingdom*

⊥*Institute for Photonics and Nanotechnologies (CNR-IFN), Piazza Leonardo da Vinci, 32, 20133 Milano, Italy*

E-mail: GuoY65@cardiff.ac.uk; BennettA19@cardiff.ac.uk

PL study for laser-written GaN before annealing

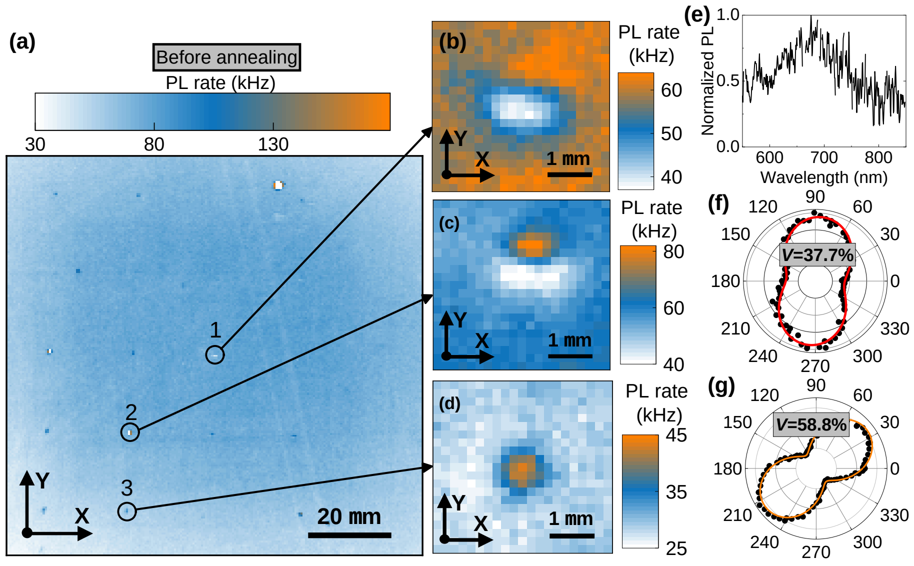

Figure S1: The PL study of laser-written GaN before any annealing process. (a) PL maps of laser-written GaN.(b-d) are the high-resolution PL maps of the laser-written areas labeled as 1-3, respectively in (a). (e) represents the spectrum of emitters in (d). (f) and (g) are the excitation polarization plots for the emitters in (c) and (d), respectively.

As shown in Fig. S1(a), the regular emitter grids have been observed from the laser-written area in the GaN substrate. The PL emission of these laser-written spots is weak relative to the background with a small dependence on the laser energy, consistent with previous studies. Some laser-written spots show a low-intensity center in the fabrication region as shown in Fig. S1(b). Some display a low-intensity center with a nearby bright emitter as shown in Fig. 4(c). These low-intensity centers are due to the laser-writing produced structural deterioration of the GaN crystal. Some laser-written areas only feature a bright emitter. The emitters in Figs. S1(c) or (d) exhibit a broad emission from 550-800 nm without a clear ZPL, and optical dipolar-like excitation polarization as shown in Fig. S1(f) and (g). The excitation polarization plots are fitted by

$$C(\theta) = A\cos^2(\theta - \theta_0) + B \tag{1}$$

where A is the amplitude, θ_0 is the polarization angle of the maximum PL rate, and B is the offset. Their visibility V of 37.7% and 58.8% for emitters in Fig. S1(c) and (d) are calculated by the

equation,

$$V = \frac{C_{\text{Max}} - C_{\text{Min}}}{C_{\text{Max}} + C_{\text{Min}}} = \frac{A}{A + 2B} \qquad (2)$$

where C_{Max} and C_{Min} are the maximum and minimum intensities, respectively.

However, no antibunching or bunching behaviors were found in their photon emission correlation spectrum (PECS). And their PL intensity scales linearly with laser power.

PL study for laser-written GaN after first 0.5 h of 400 °C annealing

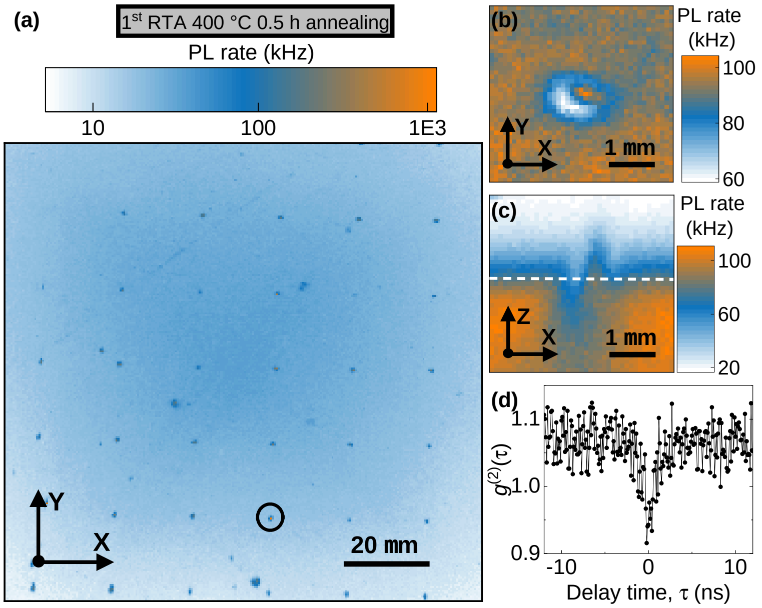

Figure S2: The PL study of laser-written GaN after the first 0.5 h of 400 °C. (a) PL maps for laser-written GaN. (b) and (c) are the x-y and x-z PL maps of the laser-written grids marked in (a), where the white dashed line represents the GaN substrate surface. (d) is the PECS of the emitters in (a-c).
